# Evaluation of the PowerScope Appliance Effects on the Dentoskeletal and Soft Tissue for the Treatment of Class II Malocclusions

**DOI:** 10.1002/cre2.70230

**Published:** 2025-10-07

**Authors:** Neda Babanouri, Roghayeh Baghandeh, Kimia Kiumarsi

**Affiliations:** ^1^ Orthodontic Research Center, School of Dentistry Shiraz University of Medical Sciences Shiraz Iran; ^2^ Department of Orthodontics School of Dentistry Shiraz University of Medical Sciences Shiraz Iran; ^3^ Student Research Committee, School of Dentistry Shiraz University of Medical Sciences Shiraz Iran

**Keywords:** class II malocclusion, functional appliance, PowerScope, Twin Block

## Abstract

**Aim:**

To evaluate and compare the dentoskeletal and soft tissue effects of the PowerScope and Twin Block appliances for treating Class II malocclusions.

**Materials and Methods:**

This retrospective study analyzed pre‐ (T0) and posttreatment (T1) cephalograms of 14 patients treated with PowerScope (Group A) and 17 patients treated with Twin Block (Group B). Thirty‐three cephalometric variables were assessed. Statistical analysis included paired and independent *t*‐tests or their non‐parametric equivalents.

**Results:**

Both groups showed significant improvements in soft tissue profiles. The Twin Block group demonstrated a significantly greater increase in SNB angle (*p* = 0.048) and more pronounced changes in UL‐SnPog' and LL‐SnPog’ posttreatment (*p* = 0.021 for both). The PowerScope group exhibited significantly greater increases in IMPA and Md‐NB (*p* = 0.029 and *p* < 0.001, respectively) and a significantly shorter treatment duration (*p* < 0.0001).

**Conclusion:**

Both appliances effectively treat Class II malocclusion and improve lip positioning. The Twin Block appliance is associated with greater mandibular skeletal advancement, while the PowerScope appliance leads to greater lower incisor protrusion and a shorter treatment time.

## 1 Introduction

Class II malocclusion is a common dental problem that may result in a range of functional and aesthetic concerns, such as chewing difficulties, speech impediments, and potential tooth wear. Timely identification and treatment can contribute to successful control of the condition, resulting in better oral health and an improved quality of life (Huo et al. 2023; Rusli et al. 2024).

Class II malocclusion treatment often involves the use of functional appliances, which can be categorized into fixed and removable types. Fixed functional appliances are permanently attached to the teeth, ensuring consistent force application without relying on patient compliance, although they can complicate oral hygiene and require regular adjustments (Moro et al. 2020). Conversely, removable functional appliances allow patients to take them out for cleaning and during meals, promoting better hygiene but relying heavily on patient adherence for effectiveness (Padmini et al. 2024). Ultimately, the choice between fixed and removable appliances should be tailored to the individual patient's needs, age, and compliance potential, with a thorough discussion with an orthodontist guiding the decision‐making process (Ngan and Tai 2024). Research indicates that both types can effectively correct overjet and improve jaw relationships, though fixed appliances generally lead to more consistent outcomes due to their non‐removable nature (Giuntini et al. 2023; Xu et al. 2024).

The Twin Block appliance, an established choice, uses bilateral occlusal blocks to achieve a similar correction but with a conventional design. Twin Block appliances have drawbacks like labial tipping of lower incisors and the requirement for patient cooperation. Conversely, fixed functional appliances like Herbst, fixed Twin Block, jasper jumper, and forsus fatigue‐resistant devices are more preferable due to patient compliance (Agarwal et al. 2024). The PowerScope appliance, a more recent device, utilizes a distinct mechanism for mandibular advancement and promotes beneficial skeletal growth patterns (Dadhwal et al. 2024). On the other hand, While both appliances target improving the occlusal relationship and enhancing facial aesthetics, their impacts on dentoskeletal structures and soft tissues can differ considerably (Subramanian et al. 2023; Pawar 2023).

There is a consensus regarding the user‐friendliness of PowerScope, which includes a short treatment duration, fast and single‐session installation, reduced bulkiness for improved aesthetics, and unrestricted jaw movement that enhances patient comfort. While recent studies and reviews have examined the PowerScope appliance (Gupta et al. 2020; Savana et al. 2016; Kalra et al. 2021), there remains a paucity of direct, comparative clinical studies evaluating its efficacy against established appliances like the Twin Block, particularly concerning their differential impacts on both dentoskeletal structures and soft tissue profiles. Therefore, this current study aims to directly compare the dentoskeletal and soft tissue effects of the PowerScope appliance with the Twin Block appliance, providing clinicians with evidence‐based insights for appliance selection. The primary hypothesis of this study is that while both appliances will effectively correct Class II malocclusion, the Twin Block appliance will produce greater skeletal (mandibular) changes, whereas the PowerScope appliance will result in more pronounced dentoalveolar changes (specifically, lower incisor protrusion) and a shorter treatment duration.

## 2 Materials and Methods

This study has received approval from the ethical committee of Shiraz Univesity of Medical Sciences (IR.SUMS.DENTAL.REC.1402.048).

### 2.1 Study Type and Samples

In this retrospective cross‐sectional study, the pretreatment (T0) and posttreatment (T1) cephalograms of 31 Class II Division 1 patients who had been treated at the orthodontic clinic of the Dental Faculty at Shiraz University of Medical Sciences from February 2018 to October 2022 were examined. Sample size determination was based on findings from previous studies, which indicated an effect size of 0.5, an *α* level of 0.05, and a power of 0.8; thus, a minimum of 14 subjects in each group had been required (Giuntini et al. 2015).

The study applied the following inclusion criteria: male and female subjects with a skeletal Class II Division 1 pattern, demonstrate a Class II canine relationship and either a full cusp or end‐to‐end Class II molar relationship, have an overjet between 5 and 10 mm, show a normal or horizontal growth pattern, exhibit 2, 3, or 4 stages of cervical vertebral maturation index (CVMI), have no history of previous orthodontic treatment, lack of any syndromic or medically compromised conditions, present no skeletal asymmetry, possess complete and available treatment documents, and achieve a Class I molar and canine relationship posttreatment. The exclusion criteria included individuals with severe crowding and misalignments, congenitally missing or extracted teeth, maxillary prognathism, and an anterior dental open bite.

### 2.2 Study Procedure

Group 1 comprises 14 patients who were treated with the PowerScope, a fixed functional appliance. The treatment regimen for this group included three phases.

**Phase 1:** Involved the leveling and aligning of the upper and lower arches using Mini Master Brackets with an MBT prescription (0.022‐in.; American Orthodontics, USA). Treatment began with a 0.014‐in. nickel–titanium (NiTi) archwire (NiTi Memory Wire; American Orthodontics, USA), followed by 0.016‐inch NiTi, 0.18‐inch stainless steel, and subsequently, a 0.019 × 0.025‐in. rectangular stainless steel archwire. The rectangular archwires were cinched behind the maxillary and mandibular molars.

**Phase 2**: Involved growth modification using the Twin Block appliance for a minimum of 16 h per day. Patient compliance was assessed clinically through regular evaluation of appliance fit, wear patterns, and patient/parental reports. Treatment progression was contingent upon achieving consistent wear and the desired quarter‐cusp Class III overcorrection of the molar and canine relationships, along with an edge‐to‐edge incisor overjet.

**Phase 3**: Involved active retention with part‐time use of Class II intermaxillary elastics for at least 1 day, over a period of 2 months. All treatment interventions in both groups were administered by the same orthodontist.

Group 2 consists of 17 patients who received treatment with the Twin Block, a removable functional appliance, in conjunction with a fixed orthodontic appliance. The treatment protocol for this group also included three phases.

**Phase 1:** Focused on leveling and aligning the maxillary arch using Mini Master Brackets with an MBT prescription (0.022‐in.; American Orthodontics, USA), concluding with a passive 0.018‐in. stainless steel archwire (Stainless Steel; American Orthodontics, USA).

**Phase 2**: Involved growth modification using the Twin Block appliance for a minimum of 16 h per day, continuing until a quarter‐cusp Class III overcorrection of the molar and canine relationships was achieved, along with an edge‐to‐edge incisor overjet.

**Phase 3:** Centered on leveling and aligning the lower arch, beginning with a 0.014‐in. nickel–titanium archwire (NiTi Memory Wire; American Orthodontics, USA). The Twin Block exhibited several modifications compared to conventional designs:

- All the lower incisors featured acrylic capping.
- The labial bow in the anterior lower segment was embedded in an acrylic bar.
- A wax relief was created on the lingual surface of the lower incisors.
- The labial bow of the upper block was removed to accommodate the fixed orthodontic braces.

### 2.3 Cephalometric Analysis

**Figure 1:**
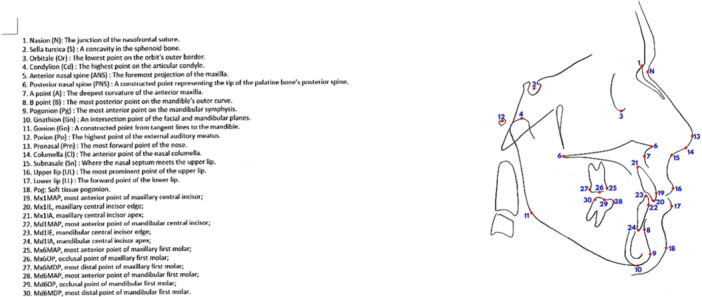
The landmarks were identified on each cephalogram, consisting of 10 skeletal landmarks, 13 dental and dentoalveolar measurements, and 10 soft tissue variables.

The lateral cephalograms were captured while in centric occlusion, with the lips relaxed. Both the initial and final lateral headfilms were traced manually. A total of 33 landmarks were identified on each cephalogram, consisting of 10 skeletal landmarks, 13 dental and dentoalveolar measurements, and 10 soft tissue variables (Figure 1).

### 2.4 Error Study

To quantify measurement errors, 10 randomly selected images from both study groups were re‐evaluated 4 weeks later by the same rater.

### 2.5 Statistical Analysis

Descriptive statistical methods, including measures of central tendency and dispersion, were employed to summarize the data, while significance tests were utilized for data analysis. The normality of the data was assessed using the Shapiro–Wilk test. For inferential analysis, independent and paired *t*‐tests were conducted, and when the data showed significant deviations from normality, non‐parametric Mann–Whitney *U* tests and Wilcoxon tests were utilized. Additionally, the *χ* ^2^ test was employed to compare gender distribution between the two groups, and the Fisher exact test was applied to evaluate the distribution of patients based on CVMI stage across groups. A significance level of 5% was established, and all analyses were conducted using SPSS software version 22.

## 3 Results

In this study, a total of 31 individuals were examined in two groups: PowerScope (*n* = 14) and Twin Block (*n* = 17). Among the participants, 61.3% (*n* = 19) were male, with 64.3% (*n* = 9) in the PowerScope group and 58.8% (*n* = 10) in the Twin Block group. This difference was not statistically significant (*p* = 0.756). Additionally, all intraclass correlation values were higher than 0.80, indicating acceptable intra‐observer agreements for all measurements.

Considering the CVM stage, in the PowerScope group, 1 patient (7.1%) was at stage 1, 11 individuals (78.6%) were at stage 2, and 2 patients (14.3%) were at stage 3. In the second group, 3 patients were at stage 1 (17.6%), 12 were at stage 2 (70.6%), and 2 patients were at stage 3 (11.8%). The results of the Fisher exact test indicated that the difference observed between the two groups was not statistically significant (*p *= 0.845). However, the PowerScope group had a statistically significantly shorter treatment duration (*p *= 0.0001) compared to the Twin Block group (Table 1).

**Table 1:** Comparison of cervical vertebral maturation index (CVMI) at the pretreatment, sex distribution, and duration of treatment in the groups.

		PowerScope group (*n* = 14)	Twin block group (*n* = 17)	*p* value
CVMI stage	I	1 (7.1%)	3 (17.6%)	0.845
	II	11 (78.6%)	12 (70.6%)	
	III	2 (14.3%)	2 (11.8%)	
Sex	Male	9 (64%)	10 (58%)	0.756
	Female	5 (36)	7 (42%)	
Duration of treatment		26.57 ± 2.19	30.58 ± 1.77	< 0.0001

### 3.1 Soft Tissue Profile Variables

The findings of the analysis for these parameters are summarized in Table 2.

**Table 2:** Comparison of soft tissue profile variables between and within two groups.

Variables	Time	PowerScope (*n* = 14)		Twin block (*n* = 17)		*p* value
		Mean	SD	Mean	SD	
Z angle	Before	61.86	9.44	60.18	6.29	0.558
	After	68.07	8.06	65.35	3.87	0.263
	Change	6.21	6.95	5.17	5.64	0.650
	*p* value	0.005		0.002		
UL‐S line	Before	0.03	2.63	−1.20	1.87	0.136
	After	1.87	1.75	0.97	1.31	0.116
	Change	1.83	1.98	2.18	2.12	0.645
	*p* value	0.004		0.001		
LL‐S line	Before	2.36	2.41	2.48	1.97	0.882
	After	0.55	3.64	0.81	1.53	0.789
	Change	−1.80	2.13	−1.66	1.87	0.843
	*p* value	0.007		0.002		
UL‐SnPog’	Before	−4.37	2.50	−3.75	2.14	0.465
	After	−3.62	1.89	−2.10	1.56	0.021*
	Change	0.75	1.83	1.64	1.21	0.114
	*p* value	0.087		< 0.001		
LL‐SnPog’	Before	−1.07	3.73	−2.52	2.54	0.207
	After	−1.45	3.56	−3.94	1.32	0.021*
	Change	−0.370	2.08	−1.41	3.06	0.084
	*p* value	0.754		0.009		
UL‐E line	Before	0.60	3.78	−1.34	2.25	0.157
	After	2.56	2.77	0.37	1.89	0.014*
	Change	1.95	1.86	1.72	1.19	0.329
	*p* value	0.002		< 0.001		
LL‐E line	Before	3.85	2.34	3.66	2.16	0.814
	After	2.19	1.95	2.01	2.22	0.814
	Change	−1.66	1.88	−1.65	1.71	0.986
	*p* value	0.006		0.001		
H‐Prn	Before	6.16	3.69	5.65	3.08	0.677
	After	7.62	3.39	7.54	3.35	0.853
	Change	1.45	2.94	1.89	2.64	0.666
	*p* value	0.087		0.009		
H.NB	Before	16.71	6.05	18.59	3.98	0.310
	After	13.57	5.37	14.24	3.23	0.674
	Change	−3.14	3.37	−4.35	2.49	0.261
	*p* value	0.004		< 0.001		
Nasolabial angle	Before	99.93	11.52	100.76	12.20	0.846
	After	104.14	10.61	106.47	6.63	0.483
	Change	4.21	8.49	5.70	9.20	0.646
	*p* value	0.086		0.021		

*Note:* Bold values and * indicate statistically significant.

#### 3.1.1 Intergroup Analysis

The statistical analysis indicated a significant intergroup difference in the posttreatment values for UL‐SnPog’ (*p* = 0.021) and LL‐SnPog’ (*p* = 0.021). However, before the intervention, these two parameters did not exhibit a significant difference between the groups, and the other variables were similar (*p* > 0.05). No additional parameters displayed statistically significant differences between the two groups.

#### 3.1.2 Intragroup Analysis

The statistical analysis indicated that within the PowerScope group, there were no significant differences in the parameters LL‐SnPog’ (*p* = 0.754), H‐Prn (*p* = 0.087), and the nasolabial angle (*p* = 0.086). Conversely, for the other parameters, the differences between pre‐ and post‐intervention measurements were significant (*p* < 0.05). In the Twin Block group, all parameters showed statistically significant differences between pre‐ and post‐intervention measurements (*p* < 0.05).

### 3.2 Skeletal Components/Growth Pattern Variables

The findings from the analysis of these parameters are summarized in Table 3.

**Table 3:** Comparison of skeletal components/growth pattern variables between and within two groups.

Variables	Time	PowerScope (*n* = 14)		Twin block (*n* = 17)		*p* value	Power
		Mean	SD	Mean	SD		
SNA	Before	80.79	3.55	80.41	2.93	0.751	0.061
	After	82.21	2.25	80.06	2.33	0.853	0.054
	Change	1.42	3.63	0.35	0.60	0.828	0.055
	*p* value	0.165		0.001			
SNB	Before	75.50	2.92	75.29	2.91	0.846	0.054
	After	78.14	2.85	79.82	2.24	0.048*	0.427
	Change	2.64	3.10	4.52	2.66	0.048*	0.515
	*p* value	0.007		< 0.001			
ANB	Before	5.57	2.76	5.35	2.26	0.810	0.056
	After	4.21	2.48	2.53	1.17	0.019*	0.670
	Change	−1.35	2.20	−2.82	2.12	0.070*	0.443
	*p* value	0.038		< 0.001			
Co‐A	Before	81.79	9.48	81.82	9.77	0.991	0.050
	After	84.30	7.46	84.47	8.61	0.954	0.050
	Change	2.51	11.31	2.64	6.43	0.968	0.050
	*p* value	0.182		0.038			
Co‐Gn	Before	98.57	6.82	97.82	6.87	0.762	0.060
	After	108.80	6.72	108.50	8.90	0.918	0.051
	Change	10.22	9.31	10.68	6.72	0.867	0.053
	*p* value	0.001		< 0.001			
Pog‐Nprep	Before	11.25	4.42	10.35	5.54	0.628	0.076
	After	8.22	5.23	6.07	5.73	0.288	0.182
	Change	−3.02	6.75	−4.28	3.01	0.495	0.103
	*p* value	0.034		0.001			
NAP	Before	7.50	4.50	8.12	3.96	0.688	0.068
	After	3.71	2.46	3.29	2.33	0.631	0.076
	Change	−3.78	4.11	−4.82	3.60	0.461	0.112
	*p* value	0.009		0.001			
SN‐OP	Before	17.00	2.82	16.94	3.75	0.962	0.050
	After	15.36	4.50	17.59	3.26	0.121	0.340
	Change	−1.64	4.58	0.64	3.74	0.136	0.317
	*p* value	0.341		0.482			
SN‐GoGn	Before	33.21	4.38	31.47	5.10	0.323	0.164
	After	32.00	32.53	−1.21	4.42	0.740	0.062
	Change	−1.21	4.42	1.05	3.84	0.137	0.316
	*p* value	0.822		0.194			

*Note:* Bold values and * indicate statistically significant.

#### 3.2.1 Intergroup Analysis

The results indicated a statistically significant difference between the two study groups concerning the change in the SNB angle (*p* = 0.048), with the Twin Block group showing a numerically greater mean change. However, the clinical significance of this difference should be interpreted with caution due to the overlap in standard deviations. A similar trend was observed in the ANB angle; there was no statistically significant difference between the two groups before the intervention (*p* = 0.810), but this difference became statistically significant after the intervention (*p* = 0.019). Additionally, the difference in observed changes approached statistical significance (*p* = 0.070). No statistically significant differences were found in other parameters between the two groups (*p* > 0.05).

#### 3.2.2 Intragroup Analysis

In the PowerScope group, the intragroup analysis revealed that changes in SNA (*p* = 0.165), Co‐A (*p* = 0.182), SN‐OP (*p* = 0.341), and CN‐Co‐Gn (*p* = 0.822) did not demonstrate significant statistical differences. However, other parameters showed significant intra‐group differences (*p* < 0.05). In the Twin Block group, the only parameters that exhibited insignificant changes pre‐ and post‐intervention were SN‐OP (*p* = 0.482) and SN‐GoGn (*p* = 0.194). For all other parameters in this group, the changes before and after the intervention were statistically significant (*p* < 0.05).

### 3.3 Dental/Dentoalveolar Component Variables

The results for the dentoalveolar parameters are summarized in Table 4.

**Table 4:** Comparison of dental/dentoalveolar components variables between and within two groups.

Variables	Time	PowerScope (*n* = 14)		Twin block (*n* = 17)		*p* value	Power
		Mean	SD	Mean	SD		
Overbite	Before	5.10	2.17	4.61	2.33	0.554	0.089
	After	2.40	1.12	1.99	0.85	0.255	0.202
	Change	−2.70	2.20	−2.62	2.10	0.922	0.051
	*p* value	< 0.001		< 0.001			
Overjet	Before	8.00	2.68	8.14	2.92	0.886	0.052
	After	3.20	1.63	2.69	1.58	0.391	0.134
	Change	−4.80	2.93	−5.45	3.11	0.556	0.089
	*p* value	< 0.001		< 0.001			
Mx1‐pp	Before	25.92	2.95	24.14	2.81	0.458	0.113
	After	27.37	3.65	27.09	3.96	0.838	0.055
	Change	1.45	2.30	1.94	3.04	0.619	0.077
	*p* value	0.035		0.018			
Mx1‐NA	Before	26.36	9.65	29.94	4.68	0.186	0.258
	After	23.14	5.50	24.53	5.42	0.487	0.105
	Change	−3.21	11.10	−5.41	3.96	0.453	0.114
	*p* value	0.299		< 0.001			
Mx1.NA	Before	7.02	2.43	7.51	1.75	0.520	0.096
	After	5.16	2.01	4.80	4.04	0.761	0.060
	Change	−1.85	3.80	−2.71	3.44	0.517	0.097
	*p* value	0.091		0.005			
Mx6‐PP	Before	20.42	3.93	20.70	3.33	0.833	0.055
	After	20.62	3.17	21.21	3.26	0.612	0.079
	Change	0.19	3.12	0.511	2.68	0.762	0.060
	*p* value	0.821		0.444			
Mx6‐Server	Before	37.53	6.99	38.17	7.51	0.809	0.056
	After	38.50	6.37	39.27	5.81	0.730	0.063
	Change	0.97	5.76	1.09	4.67	0.947	0.050
	*p* value	0.540		0.349			
IMPA	Before	104.00	8.99	110.59	5.48	0.018	0.679
	After	101.64	3.07	101.47	5.42	0.917	0.051
	Change	−2.35	9.77	−9.11	6.51	0.029*	0.604
	*p* value	0.383		< 0.001			
Md1.NB	Before	23.86	5.50	25.06	5.72	0.559	0.088
	After	32.64	2.20	27.47	4.34	< 0.001*	0.974
	Change	8.78	5.68	2.41	3.14	< 0.001*	0.969
	*p* value	< 0.001		< 0.001			
Md1‐NB	Before	5.00	1.35	5.17	1.05	0.687	0.068
	After	6.53	1.92	6.01	1.59	0.419	0.125
	Change	1.53	2.17	0.84	1.38	0.289	0.181
	*p* value	0.020		0.023			
Md1‐GoGn	Before	36.64	3.35	36.67	2.68	0.980	0.050
	After	37.80	3.37	37.70	3.45	0.940	0.051
	Change	1.15	4.04	1.03	3.63	0.930	0.051
	*p* value	0.304		0.258			
Md6‐GoGn	Before	24.85	2.37	25.50	2.26	0.448	0.116
	After	27.41	3.29	26.60	6.00	0.656	0.072
	Change	2.55	3.11	1.10	6.23	0.435	0.119
	*p* value	0.009		0.475			
Md6‐svert	Before	35.53	6.82	36.50	8.19	0.728	0.063
	After	42.55	2.88	43.79	2.13	0.127	0.267
	Change	7.01	6.23	7.29	8.51	0.919	0.051
	*p* value	0.001		0.003			

*Note:* Bold values and * indicate statistically significant.

#### 3.3.1 Intergroup Analysis

The findings revealed a significant difference between the two study groups regarding changes in IMPA and Md‐NB (*p* = 0.029 and *p* < 0.001, respectively). Both parameters increased following treatment, with the PowerScope group exhibiting greater changes. No significant differences were observed between the two study groups for changes in other dentoalveolar parameters.

#### 3.3.2 Intragroup Analysis

For the PowerScope group, a statistically significant difference was found in most parameters (*p* < 0.05), except for Mx1‐NA (*p* = 0.299), Mx1‐NA (*p* = 0.091), Mx6‐sever (*p* = 0.540), IMPA (*p* = 0.383), and Md1‐GoGn (*p* = 0.304). In the Twin Block group, statistically significant differences were noted for most parameters (*p* < 0.05), with the exception of Mx6‐pp (*p* = 0.444), Mx6‐sever (*p* = 0.349), Md1‐GoGn (*p* = 0.258), and Md6‐GoGn (*p* = 0.475).

## 4 Discussion

Early intervention in the mixed dentition stage enables orthodontists to harness the patient's growth potential for effective correction of dental and skeletal malocclusions. By influencing craniofacial growth patterns and addressing skeletal irregularities like Class III malocclusions, early orthodontic treatment enhances facial aesthetics, ensures long‐term stability, reduces treatment time, improves patient compliance, and boosts self‐esteem and confidence. The prevalence of Class II malocclusions is high and can result in functional problems and aesthetic concerns. This study sought to examine the dentoskeletal and soft tissue impacts of two different functional appliances, namely the fixed appliance PowerScope and the removable appliance Twin Block, in the management of Class II malocclusions.

The current study's findings indicate that both study groups experienced a decrease in soft tissue convexity and an improvement in the position of the upper and lower lips after the treatment period. While there was no significant difference in the amount of change between the two groups, the Twin Block group showed a slightly greater improvement in lip position when comparing the posttreatment variables. In terms of soft tissue changes, the Twin Block group exhibited more pronounced effects on upper lip positioning and lower lip positioning. The PowerScope also improved soft tissue profiles but to a lesser extent. The study highlighted that while both appliances effectively enhanced lip positioning, the Twin Block resulted in more significant changes. Furthermore, in terms of soft tissue, a significant enhancement in the facial profile was noted, attributed to an increase in the labiomental angle (Kalra et al. 2021). On the other hand, Elfeky et al. (2018) discovered a notable enhancement in facial soft tissue in terms of anteroposterior and vertical dimensions, primarily attributed to an increase in mandibular length following Twin Block therapy. This inconsistency may be due to differences in sample size and demographics. Prajwal et al. consistent with our study assess and contrast the skeletal, dentoalveolar, and soft‐tissue impacts of two functional appliances. Both groups achieved a positive impact on facial convexity. The PowerScope group showed a notable growth in the dimensions of the upper and lower pharynx. Both groups exhibited a statistically significant reduction in upper lip protrusion, an increase in lower lip protrusion, an enhanced nasolabial angle, and a decrease in the inferior labial sulcus (Pawar 2023).

The study found that the Twin Block appliance led to a statistically significant increase in the SNB angle compared to the PowerScope group, suggesting a trend towards greater mandibular advancement. The clinical relevance of this difference, however, warrants careful consideration given the variability in individual responses. Specifically, the change in the SNB angle was statistically significant for the Twin Block group, while changes in other parameters like SNA and Co‐A did not show significant differences between the two groups. However, both appliances were effective in reducing the ANB angle, which reflects improvements in occlusal relationships. Kalra et al. (2021) reported that statistically significant alterations were observed in skeletal parameters, including the forward positioning of the mandible, which was indicated by an increase in the SNB angle and the N perpendicular–Pogonion distance. Additionally, there was an improvement in the Class II jaw base relationship, evidenced by a reduction in the ANB angle and Wits appraisal. The Twin Block appliance showed a tendency for greater mandibular advancement during treatment. This appliance encourages mandibular lengthening by stimulating increased growth at the condylar cartilage when adjusted to a protrusive bite, utilizing the occlusal inclined plane as a guiding mechanism, which advances the mandible forward. Several studies have evaluated the impact of the Twin Block and Frankal appliances on dental and skeletal structure. However, there has been no investigation into the effectiveness of fixed functional appliances like Forsus and PowerScope in the treatment of Class II malocclusion when compared to the Twin Block and Frankal appliance (Weiland and Bantleon 1995). While both functional appliances effectively addressed the Class II malocclusion, there were minor differences in the skeletal and dentoalveolar changes observed. In our study, both Twin Block and PowerScope significantly increased the mandibular length; however, no significant difference was observed between the two groups. In a similar manner to our investigation, Toth et al. (1999) observed a decrease in ANB angle, Sharma et al. (2012) noted a reduction, and Khoja et al. (2016) identified a decrease in ANB angle after the implementation of Twin Block, leading to an enhancement in the skeletal relationship between the mandible and maxilla. Unlike PowerScope, this study revealed that Twin Block significantly lowered SNA, indicating a notable restriction in the anterior growth of the maxilla. The forward positioning of the mandible by the Twin Block device results in a counteracting force exerted distally on the maxilla, thereby impeding its forward growth (headgear effect) (Sharma et al. 2012).

The maxillary incisors exhibited increased lingual tipping and retrusion as a result of the force applied by the Twin Block appliance. Conversely, the lower incisors showed more prominent protrusion and labial tipping after treatment with the PowerScope appliance. The positioning of the lower incisors is crucial in Class II correction with functional appliances. Excessive labial tipping of the lower incisors should be minimized as it diminishes the potential for orthopedic change (Sharma et al. 2012). The significant increase in mandibular incisor proclination with the PowerScope may be attributed to the telescopic mechanism of the appliance, which applies a mesially directed force on the mandibular incisors (Malhotra et al. 2018; Manni et al. 2012; Elkordy et al. 2016). In both study groups, there was no alteration in the mesiodistal and vertical position of the maxillary first molar. This result aligns with the findings of Mills and McCulloch (1998) and Clark (2014) after the use of Twin Block appliances. Despite the typical distal and intrusive force exerted by functional appliances on maxillary molars, our study did not observe clinically significant distalization and intrusion, similar to the study by Malhorta et al. (2018) on the impact of PowerScope appliance. On the other hand, another study noted a distal movement of the maxillary dentition in the PowerScope group (Arora et al. 2018). Other study found no clinically significant distalization or intrusion of the maxillary molars when using the PowerScope appliance, consistent with my findings (Malhotra et al. 2018). In contrast, Kalra et al. (2021) documented a notable distalisation and intrusion of the maxillary molar following a 5‐month application of PowerScope. Differences in measurement techniques used across studies can result in discrepancies in reported outcomes.

The mandibular molars in both study groups exhibited mesial and occlusal movement on the opposite side. Lower molars showed a tendency for increased extrusion when treated with the Powerscop appliance. These results are attributed to the downward and forward force applied to the mandibular teeth (Arora et al. 2018). Antony et al. (2018) found that the PowerScope appliance led to significant mesial movement of the mandibular molars, with insignificant extrusion. Likewise, Malhorta et al. (2018) noted extrusion and mesial displacement of mandibular molars after a 6‐month application of PowerScope. Additionally, another study documented mesial migration and slight extrusion of the mandibular molars during functional appliance therapy, further corroborating the findings on the PowerScope appliance (Baccetti et al. 2005). There was not any rotation in the occlusal plan following treatment in both study groups. In the Twin Block group, there was only an insignificant increase in the cant of the occlusal plane which can be attributed to the very negligible supra eruption of mandibular molars. This finding was in accordance with the study conducted by Mills and McCulloch (1998) and Johnson et al. (2021) following treatment with Twin Block. This finding is in contrast with the results of Malhorta et al. (2018) who reported clockwise rotation of the occlusal plane after 6 months of treatment with PowerScope.

## 5 Limitations

The study has several limitations that should be considered when interpreting the results. First, the relatively small sample size may limit the statistical power and generalizability of the findings to larger populations. Additionally, the retrospective cross‐sectional design was chosen for feasibility, as a prospective study would have required a significantly longer timeframe for patient recruitment and treatment. While this design allowed us to utilize high‐quality, standardized records from a single operator, it inherently relies on pre‐existing data, which introduces potential selection bias and makes it challenging to establish causality. Another limitation is the variability in CVMI stages among participants, which could influence the response to treatment despite the lack of statistically significant differences between groups. Lastly, the study focused only on short‐term outcomes and did not include long‐term follow‐up, leaving questions about the stability and sustainability of the observed dentoskeletal and soft tissue changes unanswered. These limitations highlight the need for future research with larger sample sizes, prospective designs, and longer follow‐up periods to validate the findings.

## 6 Conclusion

In conclusion, there was an enhancement in convexity and lip positions of soft tissue following treatment in both groups. Additionally, differences were noted in skeletal and dentoalveolar changes between the two functional appliances, with the Twin Block demonstrating more significant mandibular advancement. Changes in incisors were also evident, as the Twin Block resulted in lingual tipping and retrusion of maxillary incisors, whereas the PowerScope led to labial tipping and protrusion of lower incisors. It is crucial for orthodontists to thoroughly evaluate individual cases, considering both skeletal and dental changes, as well as patient‐specific aesthetic goals when selecting a functional appliance. Future research could further elucidate the long‐term outcomes associated with each appliance type, examining not only the immediate positional changes but also their impacts on overall occlusion stability, periodontal health, and patient quality of life.

## Author Contributions

Data collection: Neda Babanouri, Kimia Kiumarsi, and Roghayeh Baghandeh. Data analysis: Neda Babanouri, Kimia Kiumarsi, and Roghayeh Baghandeh. Manuscript revision: Neda Babanouri, Kimia Kiumarsi, and Roghayeh Baghandeh. Manuscript writing: Neda Babanouri and Roghayeh Baghandeh. Statistical analysis: Kimia Kiumarsi and Roghayeh Baghandeh.

## Conflicts of Interest

The authors declare no conflicts of interest.

## Data Availability

All data have been presented in the manuscript.
